# Microbial Communities in Changing Aquatic Environments

**DOI:** 10.3390/microorganisms12040726

**Published:** 2024-04-03

**Authors:** Damir Kapetanović, Mohammad Katouli, Darija Vukić Lušić

**Affiliations:** 1 Ruđer Bošković Institute, 10000 Zagreb, Croatia; 2 Centre for Genecology, School of Health and Sport Sciences, University of the Sunshine Coast, Sippy Downs, QLD 4556, Australia; mkatouli@usc.edu.au; 3 Faculty of Medicine, University of Rijeka, 51000 Rijeka, Croatia; darija.vukic.lusic@medri.uniri.hr

The quality of aquatic ecosystems is an important public health concern [1]. The assessment and management of an aquatic ecosystem, including water and sediment [2], and the ecological monitoring of the microorganisms found there can provide information on the state of the environment and water quality in order to protect human and animal health. The microbial community is an essential component of aquatic ecosystems, in which heterotrophic bacteria play an important role in the biodegradation and transformation of organic matter and in the self-purification of water bodies. To be safe for consumption, water must be free of pathogenic bacteria, of which enteric pathogens are the most common [3]. In this sense, data on the microbial quality of freshwater and marine ecosystems under anthropogenic influence are of great importance for the management of water bodies. This is particularly important when drinking water resources are at stake [4,5]. Understanding the microbial communities in water provides insight into the ecological niches occupied by pathogens [6,7]. That improves our ability to recognize them early to reduce the risk to human and animal health. The aquatic microbiota plays a fundamental role in many biogeochemical processes [8]. Environmental changes and responses to temperature changes due to climate change could promote the growth of opportunistic pathogens. The composition, determinants, co-occurrence pattern, and assembly mechanism of the bacterial community define responses to changes in numerous environmental factors [9]. These are, for example, temperature, salinity, pH, dissolved oxygen concentration, availability of nutrients, organic matter, light, and pressure [4,9]. Particular attention is paid to opportunistic pathogens such as *Vibrio* [10,11], *Legionella* [12], *Listeria* [13], etc., which can multiply in altering surroundings and develop multiple resistances. Due to the weakening of immunity in the population, more and more people are affected. The study of catabolic potential enables a better understanding of the taxonomic relationships and ecological role of certain microorganisms [14]. Research is focusing on the profile of the microbiota, which is responsible for the degradation of various contaminants that are increasingly polluting the environment. Novel methods such as MALDI TOF MS, phylogenetic analyses of single and multiple genes, metagenomic sequencing technologies, large-scale ecosystem studies using DNA metabarcoding, and inductively coupled plasma-optical emission spectrometry (ICP-OES) help us better characterize the aquatic microbiota. Despite the long tradition of research in microbiology as well as the application of different research methods, we encounter gaps in knowledge about this topic. Therefore, these 12 papers have brought new insights into the study of microbial communities in changing aquatic environments. As expected, the effects of changing environmental conditions on microbial communities were observed locally and at the micro-level, for example, in the part of the water supply system of interest but also in larger aquatic ecosystems such as riverine and marine ecosystems. This allows us to gain a deeper understanding of the adaptation mechanisms of microorganisms to the changing ecosystem. For the future, the challenge remains to combine data from novel tools on the composition and dynamics of different microbial communities with basic, traditional methods. This will make it possible to create new tools for the successful management of water resources in an environment that is changing rapidly under the influence of human activities and climate change.
